# Vaginal misoprostol versus intracervical Foley catheter for cervical ripening in postdate primigravid women: a randomized clinical trial

**DOI:** 10.1186/s12884-021-04011-0

**Published:** 2021-07-27

**Authors:** Nazanin Abdi, Azin Alavi, Forough Pakbaz, Hossein Darabi

**Affiliations:** 1 Fertility and Infertility Research Center, Hormozgan University of Medical Sciences, Bandar Abbas, Iran; 2 The Persian Gulf Tropical Medicine Research Center, The Persian Gulf Biomedical Sciences Research Institute, Bushehr University of Medical Sciences, Bushehr, Iran

**Keywords:** Postdate pregnancy, Misoprostol, Foley catheter, Cervical ripening

## Abstract

**Background:**

Being one of the most common indications of labor induction, postdate pregnancy can lead to serious maternal and fetal complications. In this study we aimed to compare vaginal misoprostol with intracervical Foley catheter (FC) for cervical ripening in postdate primigravid women.

**Methods:**

This randomized clinical trial included 120 primigravid women aged 18–35 years with singleton,  postdate pregnancies, and Bishop score ≤ 4. Participants were randomized into two equal groups. The first group received 25 µg vaginal misoprostol and the second group had an 18 Fr FC inserted into their cervical canal. Labor induction was performed using oxytocin in both groups if progression of labor or true contractions did not occur within 6 h of the interventions. In case of nonreassuring fetal heart rate, fetal distress, placental abruption, or prolonged labor, C-section was performed.

**Results:**

The frequency of normal vaginal delivery, Cesarean section, meconium-stained amniotic fluid, and neonatal intensive care unit admission did not differ significantly between groups. Placental abruption and uterine tachysystole occurred more frequently in the misoprostol group (15.0 vs. 1.7%, *P* = 0.008 and 21.7 vs. 0.0%, *P* < 0.001, respectively). A significantly higher number of women in the FC group required oxytocin (73.3 vs. 41.7%, *P* < 0.001). Duration of labor was significantly higher in the FC group (*P* = 0.001).

**Conclusions:**

Due to the lower rate of placental abruption and uterine tachysystole observed with FC, it appears to be superior to vaginal misoprostol for cervical ripening in postdate primigravid women; however, its longer labor duration and higher oxytocin requirement should be taken into consideration.

**Trial registration:**

Iranian Registry of Clinical Trials, IRCT20181218042033N4. Registered 19/04/2020. Retrospectively registered, https://www.irct.ir/trial/47037

## Introduction

Postdate pregnancy, defined as extension of pregnancy to over 40 weeks of gestation, accounts for 5–14% of all deliveries across different studies [1–3]. Its etiology is still unknown; however, genetic predisposition, history of postdate pregnancy, fetal anomalies, maternal obesity, male fetus, fetal adrenal insufficiency, placental sulfatase deficiency, and primiparity have been proposed as potential risk factors [3]. Postdate pregnancy is associated with an increased possibility of maternal and fetal adverse outcomes, including fetal asphyxia, meconium passage and aspiration, low Apgar score, umbilical cord compression, abnormal fetal pulse, macrosomia, dystocia, maternal perineal lacerations, infection, postpartum hemorrhage, instrumental delivery, and cesarean section (C-section) [4–7]. Moreover, it has been reported that perinatal mortality significantly increases after 41 weeks compared to 40 weeks [8].

Postdate pregnancy is among the most common indications of labor induction whose success depends upon the degree of cervical ripening evaluated by the Bishop score [9]. Cervical ripening which is the process of softening and stretching of the cervix, is a prerequisite for labor induction, in that an unripe cervix with a low Bishop score significantly increases the risk of induction failure compared to a favorable cervix [10]. A variety of mechanical and pharmacological methods have been used for this purpose [11]. One of the most common mechanical methods of cervical ripening is the insertion of a Foley catheter (FC) into the cervical canal. Intracervical FC contributes to cervical ripening directly by dilation of the cervix and indirectly by stimulation of cytokines, prostaglandin, and oxytocin secretion [12]. Due to a twofold increased risk of infection with FC, it is contraindicated in pregnant women with apparent infection [13]. Although this method is generally endured well by women, it may result in discomfort, pain, anxiety, and mild bleeding; nevertheless, it is mostly considered efficacious and safe [11].

Misoprostol is a synthetic analogue of prostaglandin E_1_ (PGE_1_), primarily registered for the treatment and prevention of peptic ulcer disease [14]. Its many advantages include cost-effectiveness, stability at room temperature (no need for refrigeration), availability in various forms and dosages, and applicability through different routes such as vaginal, oral, buccal, and sublingual [15, 16]. Nonetheless, uterine hyperstimulation, neonatal asphyxia, and higher cesarean delivery can occur with different forms of misoprostol [16, 17].

Given the high prevalence of postdate pregnancy and the associated risks and complications, the need for an effective, inexpensive, and readily available method for cervical ripening in this population, and the inconsistency regarding the findings of previous studies as to which method is superior, we aimed to compare vaginal misoprostol with intracervical FC for cervical ripening in postdate primigravid women.

## Methods

### Participants

In this randomized clinical trial, we recruited 120 primigravid women aged 18–35 years with postdate pregnancies and Bishop score ≤ 4 from Shariati Hospital, Bandar Abbas, Iran, from August 15, 2017 to March 18, 2018. Considering the mean delivery time in the vaginal misoprostol group (11.08 ± 5.6 h) compared to the FC group (13.6 ± 16.0 h) of the study by Roudsari et al. [18], as well as α = 0.05 and β = 0.2, the sample size was calculated as at least 114 (57 participants in each group). In order to increase the power, we included a total of 120 patients (60 in each group). Shariati Hospital is an obstetrics and gynecology center. The annual rate of deliveries in the aforementioned period was around 6000, of which approximately 70% were normal vaginal deliveries (NVDs) while the rest were C-sections.

Inclusion criteria were singleton living pregnancy and vertex presentation. Exclusion criteria were any evidence of active labor or intrauterine infection, uterine scarring, hypersensitivity to misoprostol, gestational age (GA) < 41 weeks, any systemic comorbidity (preeclampsia, diabetes, cardiovascular disease, kidney disease, asthma, etc.), fetal macrosomia, nonreassuring fetal heart rate in nonstress test (NST), polyhydramnios, intrauterine growth retardation, fetal malpresentation, dystocia, premature rupture of membranes (PROM), unfavorable pelvis for normal vaginal delivery (NVD) or any other contraindication for NVD, vaginal bleeding, and placenta previa.

### Study design

GA was determined for all women using the first trimester ultrasound. Maternal age was recorded for every participant. Also, body mass index (BMI) was calculated for each patient by dividing her weight (kg) by the square of her height (m). Participants were randomized into two equal groups using block randomization. Sixty women in the misoprostol group received 25 µg vaginal misoprostol and fetal heart rate was closely monitored. Each participant of this group only received a single 25 µg dose of vaginal misoprostol. An 18 Fr FC was inserted into the endocervical canal of the 60 women in the FC group. Once past the internal os of the uterus, FC was fixed using 30 ml of sterile water injected into the FC balloon. Traction was then applied by taping the end of the catheter to the medial aspect of the women’s thigh. If progression of labor or true labor contractions did not occur within 6 h of the intervention in any of the groups, labor induction would be performed using oxytocin. The course of labor was monitored and in case of nonreassuring fetal heart rate, fetal distress, placental abruption, or prolonged labor, C-section was performed; otherwise, natural progression of labor was allowed.

The frequency of NVD, C-section, neonatal intensive care unit (NICU) admission, meconium-stained amniotic fluid (AF), placental abruption, oxytocin requirement, uterine tachysystole, and duration of labor were recorded and compared between groups. Placental abruption was diagnosed based on the presence of vaginal bleeding, uterine tenderness, and postpartum placental hematoma. However, all cases of placental abruption in this study only had placental hematoma upon clinical examination of the placenta after delivery. Moreover, birth weight as well as 1- and 5-min APGAR scores of neonates were recorded.

### Data analysis

The Statistical Package for the Social Sciences (SPSS) software (version 25.0, Armonk, NY: IBM Corp.) was used for data analysis. Mean, standard deviation, frequency, and percentages were used to describe the results. Chi-squared and Fisher’s exact tests were used to compare frequencies between groups. Student’s t-test was used to compare quantitative data between groups. P-values ≤ 0.05 were regarded as statistically significant. Risk estimation was done using odds ratio (OR) and 95% confidence intervals (CIs) were calculated. CIs that crossed 1 implied a significant difference between the study groups.

## Results

The misoprostol and FC groups were similar regarding maternal age, gestational age, body mass index (BMI), birth weight, and 1- and 5-min APGAR scores (Table 1).

**Table 1:** Comparison of baseline study variables between the misoprostol and FC groups

Variable	Misoprostol group (*N* = 60)	FC group (*N* = 60)	*P*-value^a^
Maternal age (years) mean ± SD	27.35 ± 5.44	29.47 ± 6.21	0.235
GA (weeks) mean ± SD	42.37 ± 2.13	42.77 ± 4.73	0.097
Maternal BMI (kg/m^2^) mean ± SD	29.29 ± 11.17	30.11 ± 9.62	0.548
Birth weight (g) mean ± SD	3420.34 ± 101.62	3112.57 ± 81.54	0.241
1-min APGAR score, mean ± SD	9.25 ± 0.56	9.46 ± 1.01	0.333
5-min APGAR score, mean ± SD	9.74 ± 1.22	9.69 ± 0.26	0.333

*Abbreviations:* *N* number, *SD* standard deviation, *FC* Foley catheter, *GA* gestational age, *BMI* body mass index, *APGAR* Appearance, Pulse, Grimace, Activity, and Respiration

^a^ Analyzed by Student’s t-test

Table 2 demonstrates the comparison of the main study variables between the misoprostol and FC groups. From the 120 primigravid women evaluated in the current study, 95 (79.2%) delivered their babies through NVD and 25 (20.8%) with C-section. The frequency of NVD and C-section were comparable between the misoprostol and FC groups (73.3 vs. 85.0%, *P* = 0.116 and 26.7 vs. 15.0%, *P* = 0.116, respectively). Also, the frequency of meconium-stained AF and NICU admission did not differ significantly between groups (*P* = 0.432 and *P* = 1.000, respectively). However, a significantly higher rate of placental abruption and uterine tachysystole was observed in the misoprostol group (*P* = 0.008 and *P* < 0.001, respectively). On the other hand, significantly more women in the FC group required oxytocin compared to the misoprostol group (*P* < 0.001). Furthermore, duration of labor was significantly higher in the FC group (*P* = 0.001). Of note, no perinatal death occurred in any of the groups.

**Table 2:** Comparison of the main study variables between the misoprostol and FC groups

Variable	Misoprostol group (N = 60)	FC group (N = 60)	Total	*P*-value	OR	95% CI
NVD N (%)	44 (73.3)	51 (85.0)	95 (79.2)	0.116^a^	0.49	0.19–1.21
C-section N (%)	16 (26.7)	9 (15.0)	25 (20.8)	0.116^a^	2.06	0.83–5.12
Placental abruption N (%)	9 (15.0)	1 (1.7)	10 (8.3)	0.008^a^	10.41	1.28–85.00
Uterine tachysystole N (%)	13 (21.7)	0 (0.0)	13 (10.8)	< 0.001^a^	2.28	1.84–2.82
Meconium-stained AF N (%)	10 (16.7)	7 (11.7)	17 (14.2)	0.432^a^	1.51	0.54–4.29
Oxytocin requirement N (%)	25 (41.7)	44 (73.3)	69 (57.5)	< 0.001^a^	0.26	0.12–0.56
NICU admission N (%)	3 (5.0)	2 (3.3)	5 (4.2)	1.000^b^	1.53	0.25–9.48
Labor duration (minutes) mean ± SD	1425.67 ± 69.98	2023.92 ± 201.57		0.001^c^		

*Abbreviations:* *N* number, *SD* standard deviation, *OR* odds ratio, *CI* confidence interval, *NVD* normal vaginal delivery, *C-section* Cesarean section, *AF* amniotic fluid, *NICU* neonatal intensive care unit

^a^Analyzed by Chi-squared test

^b^Analyzed by Fisher’s exact test

^c^Analyzed by Student’s t-test

Women in the misoprostol group had almost a 10.5-fold higher risk of placental abruption and approximately two-fold higher risk of uterine tachysystole compared to the FC group. Nevertheless, the odds of oxytocin requirement in the misoprostol group was nearly one fourth the odds of oxytocin requirement in the FC group (Table 2).

## Discussion

In the presence of an unfavorable cervix, induction of labor can increase the possibility of prolonged labor and incidence of C-section [19]. Therefore, different methods have been used for cervical ripening prior to labor induction. To the best of our knowledge, our research is the second study comparing FC with vaginal misoprostol for cervical ripening in postdate primigravid pregnancies since the study performed by Kandil et al. in 2010 [20].

We found that uterine tachysystole occurred less frequently with FC while significantly higher oxytocin requirement and labor duration were observed with FC compared to vaginal misoprostol. Moreover, the risk of C-section, NICU admission, and AF meconium staining, was lower in the FC group compared to the misoprostol group, but not at a statistically significant level. Kandil et al.’s findings were similar with respect to the higher need for oxytocin augmentation in the FC group; however, contrary to our study, they found a significanlty shorter  induction-delivery interval with FC [20]. The discrepancy between the two studies can be explained by the difference in the study designs, the sample size, and the demographic characteristics of the participants. In addition, Kandil et al. reported no NICU admission in either of the groups [20]. Furthermore, consistent with our results, Noor et al. found no significant difference in NICU admission between the misoprostol and FC groups [19].

In our study, the rate of placental abruption was significantly higher with vaginal misoprostol (15%) compared to FC (1.7%). Previous studies have reported various rates of placental abruption with misoprostol. Placental abruption occurred in none of the patients in the misoprostol group and only 1 patient in the FC + oxytocin group of the study by Filho et al. [21]. Balci et al. reported placental abruption in 1 patient in the misoprostol + oxytocin group of their study [22]. Fontenot et al. showed a significantly higher rate of placental abruption with misoprostol (13.7%) compared to dinoprostone (1.9%) [23]. The higher rate of placental abruption in our study can be due to the difference in definition. The cases of placental abruption in the current study were diagnosed postpartum, when we observed placental hematoma after delivery by clinical examination of the placenta. We know that none of the cases leading to C-section were because of placental abruption; however, we cannot determine whether any of these cases of abruption had caused NICU admission or fetal distress. This questions the clinical significance of the high rate of placental abruption in the misoprostol group of our study.

Garba et al. also conducted a study on postdate pregnancies, reporting a significantly shorter induction-delivery interval in the misoprostol group and comparable maternal and neonatal outcomes in both groups [24]; however, women of their study were all multigravida and oxytocin was synchronously infused in the FC group, which makes it different from the current study.

Noor et al. conducted a study on women with term gestation comparing 25 µg vaginal misoprostol given every 4 h to 16 Fr FC inflated with 50 ml of sterile saline [19]. Their results regarding the induction to delivery interval and uterine hyperstimulation were in line with our findings; nevertheless, the rate of NVD was significantly higher in the misoprostol group of their study. Their study included both primigravid and multigravida term pregnancies with different indications for labor induction, while we only recruited primigravid women with postdate pregnancy as an indication for labor induction, which may be the reason for the difference between their results and ours. Also, contrary to our findings, Tuuli et al. reported no significant difference in the total duration of labor in the misoprostol group compared to the FC group [25]. The shorter duration of labor in the misoprostol group our study can be justified by the greater effect of misoprostol due to direct delivery to myometrium through cervical canal via the vaginal route.

Gondkar et al. suggested equal efficacy and safety of FC and vaginal misoprostol for labor induction [26]. Similarly, Fox et al. found FC and vaginal misoprostol to be equally effective as induction agents [27]. Nonetheless, in this meta-analysis of 1603 patients, the rate of uterine tachysystole was significantly higher in patients receiving misoprostol compared with women receiving transcervical FC, which is consistent with our results. The lower rate of tachysystole with FC is particularly important in patients at increased risk of fetal hypoxia, such as those with postdate pregnancy since varying degrees of placental insufficiency may be present in this population. However, the overall higher incidence of tachysystole in our misoprostol group (21.7%) compared to Filho et al.’s study (1.7%) [21] and Kandil et al.’s research (0%) [20], considering that they used higher doses of misoprostol (25 µg every 6 h and 25 µg every 4 h, respectively) can be justified by the earlier use of oxytocin in our study after 6 h, which may have caused tachysystole in the vaginal misoprostol group by adding to the effect of misoprostol.

In accordance with our findings, Jozwiak et al. demonstrated that oxytocin is significantly more often required when FC is used [28]. As they suggested, this can be interpreted into the inability of FC to cause contractions. In their opinion, FC can merely ripen the cervix, an advantage for cases of intolerability for contractions including intrauterine growth retardation of oligohydramnios [28].

Some studies have investigated the effect of FC combined with vaginal misoprostol. As a matter of fact, the use of intracervical FC plus vaginal misoprostol has been compared with vaginal misoprostol alone in a very recent meta-analysis. In this study, Lee et al. showed that induction time, uterine tachysystole, and meconium staining decrease with the combination of FC and vaginal misoprostol compared to misoprostol alone with no difference regarding the C-section rate [29].

One limitation of the current study was the impossibility of blinding due to the nature of the interventions, which can make the assessment of outcomes prone to bias. Another limitation of our study was that we did not take infections into account. FC insertion has been reported as a risk factor for chorioamnionitis in a recent meta-analysis [30]. Besides, in the only other study performed on postdate primigravid women, prophylactic ampicillin was administered in the FC group to prevent infection [20]. Various volumes have been used to fill the FC balloon across different studies and some studies suggest that higher volumes are more effective for labor induction [31, 32]. We might have achieved better results regarding the rate of NVD in the FC group if we had used 50 ml of sterile saline for FC balloon inflation instead of 30 ml.

## Conclusions

The present study suggests that in postdate pregnant women with viable singleton gestation, FC appears to be superior in terms of lower incidence of placental abruption and uterine tachysystole without increasing the risk of meconium-stained AF, C-section, and NICU admission. However, vaginal misoprostol is associated with shorter labor duration and less oxytocin requirement. Adequately powered studies are required to confirm the findings of the current study.

## Data Availability

The datasets used and/or analyzed during the current study are available from the corresponding author on reasonable request.

## Abbreviations

AF: Amniotic fluid
APGAR: Appearance, Pulse, Grimace, Activity, and Respiration
BMI: Body mass index
CI: Confidence interval
C-section: Cesarean section
FC: Foley catheter
GA: Gestational age
IRCT: Iranian Registry of Clinical Trials
NST: Nonstress test
NVD: Normal vaginal delivery
OR: Odds ratio
SPSS: Statistical Package for the Social Sciences
